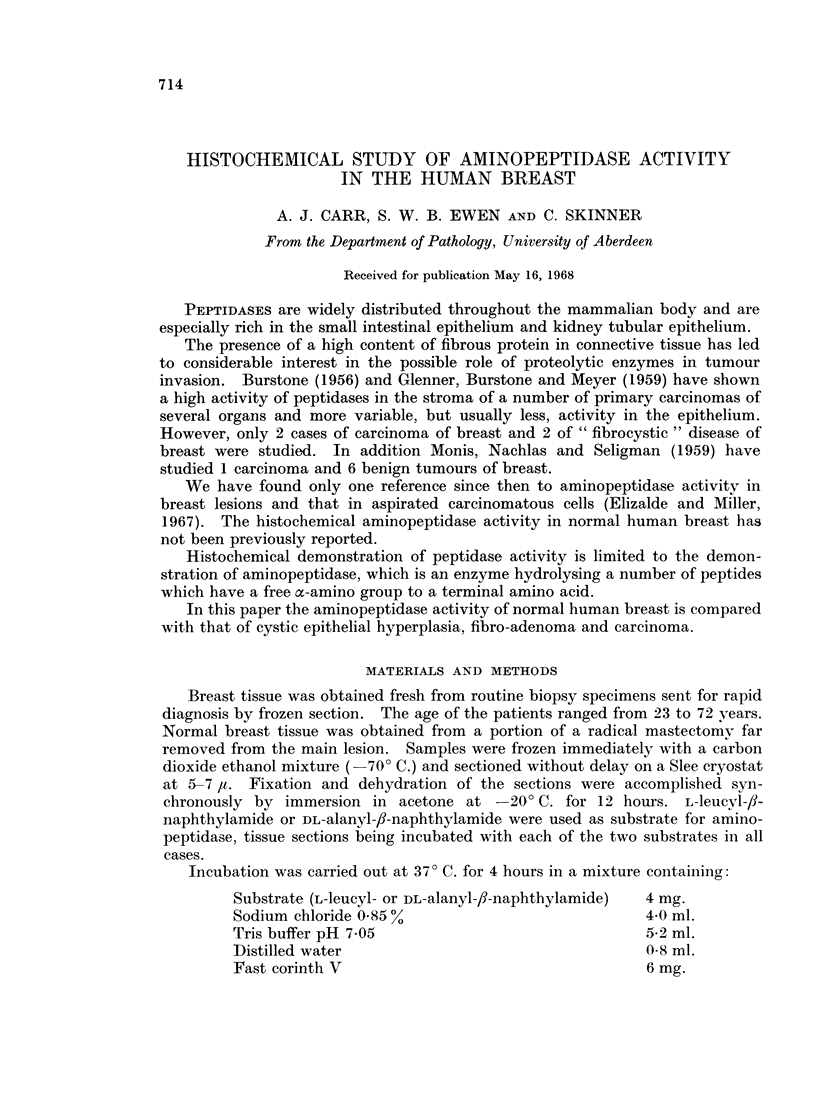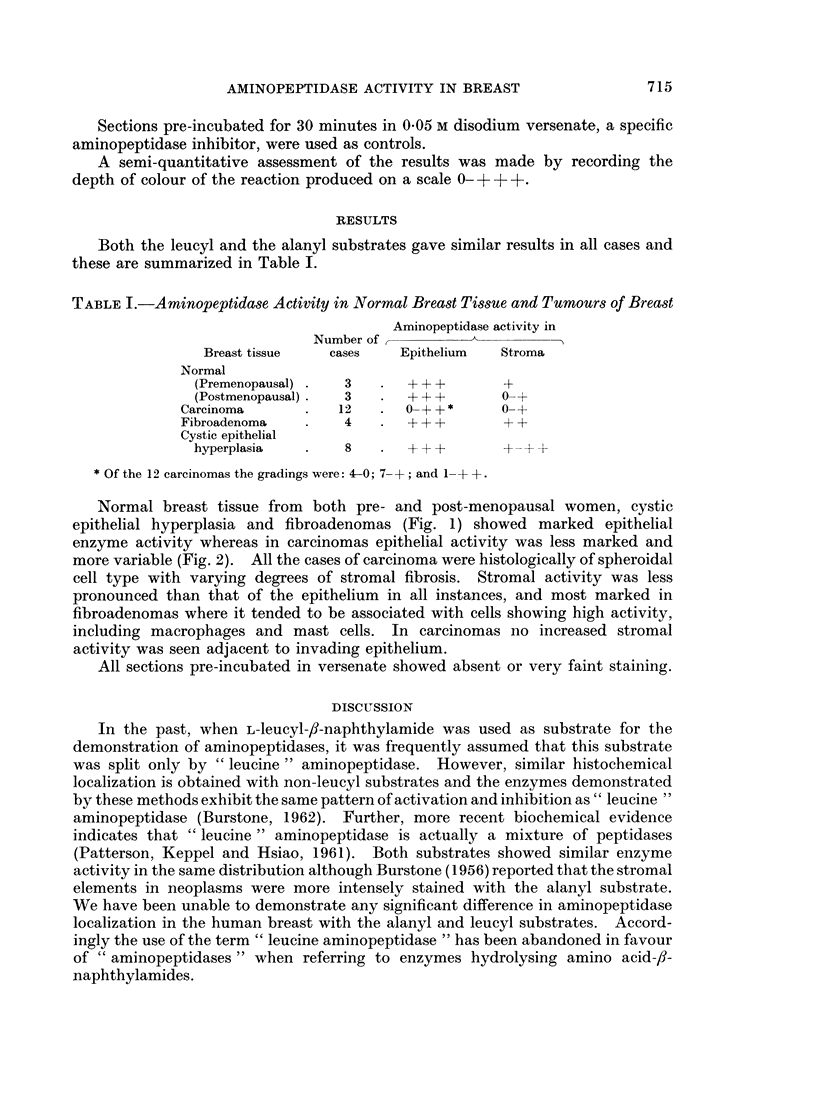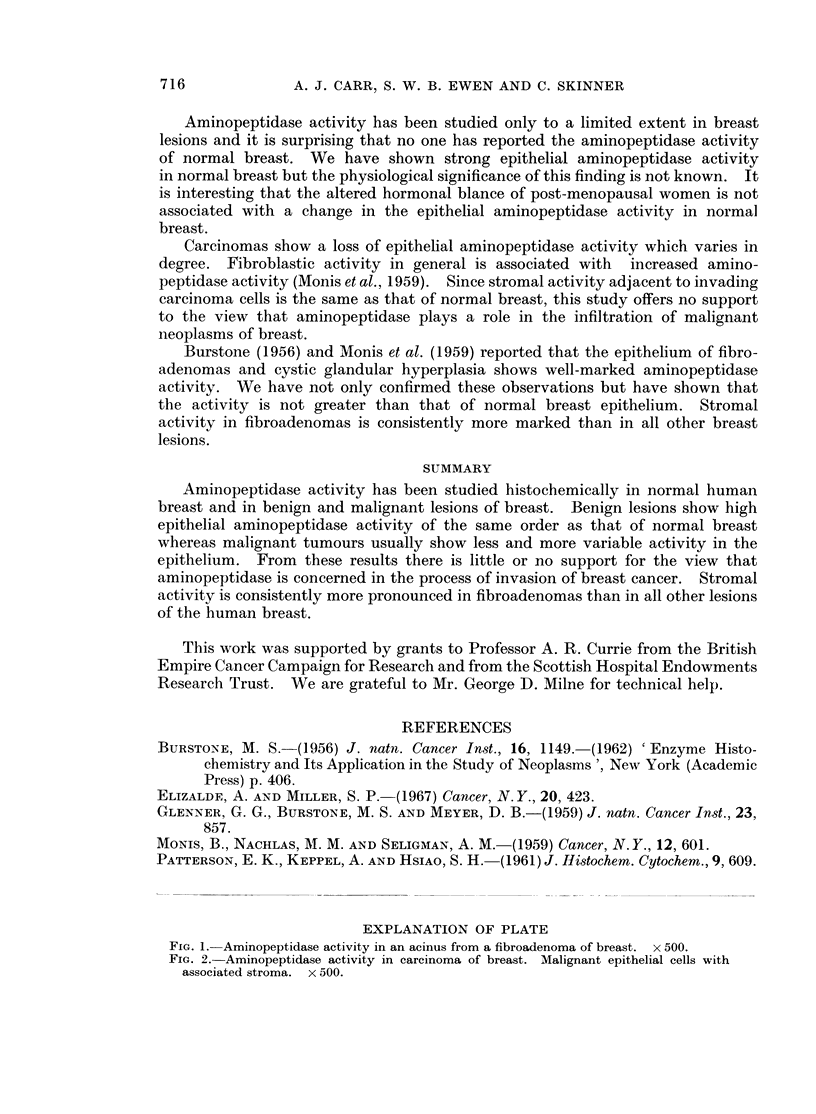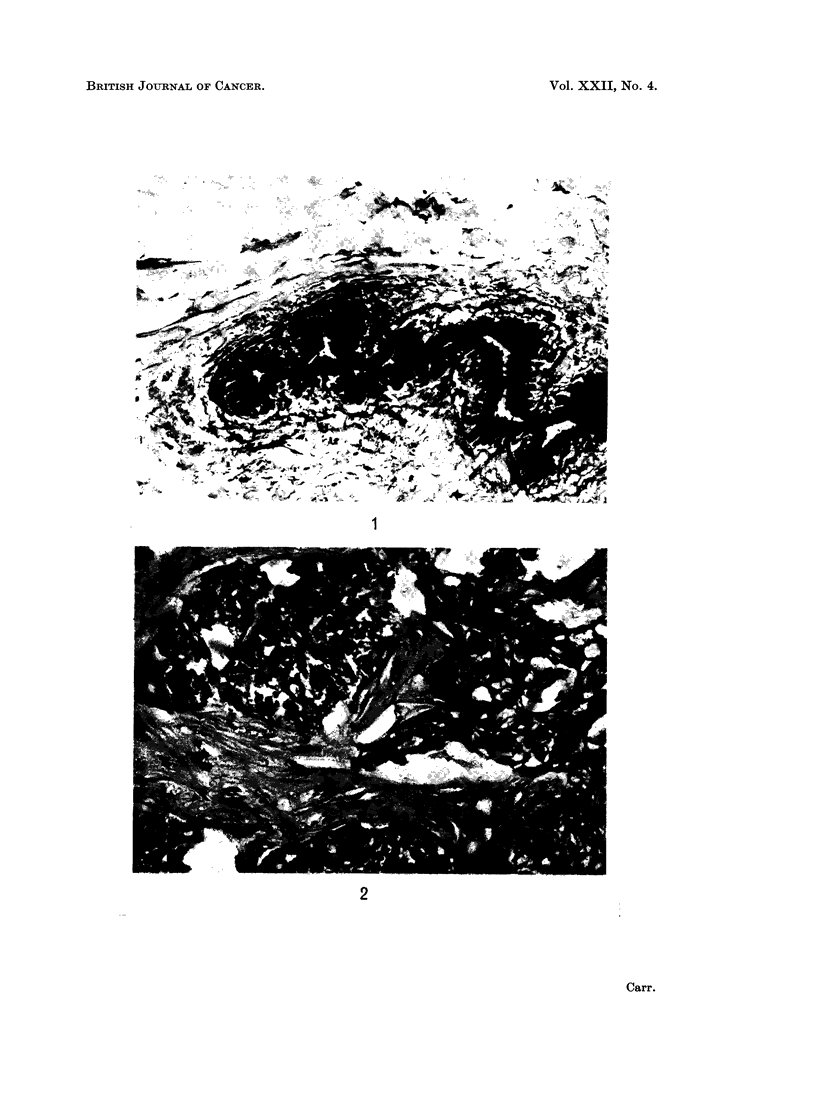# Histochemical study of aminopeptidase activity in the human breast.

**DOI:** 10.1038/bjc.1968.84

**Published:** 1968-12

**Authors:** A. J. Carr, S. W. Ewen, C. Skinner

## Abstract

**Images:**


### HISTOCHEMICAL STUDY OF AMINOPEPTIDASE ACTIVITY IN THE HUMAN BREAST

A. J. CARR, S. W. B. EWEN AND C. SKINNER

*From the Department of Pathology, University of Aberdeen*

Received for publication May 16, 1968

PEPTIDASES are widely distributed throughout the mammalian body and are especially rich in the small intestinal epithelium and kidney tubular epithelium.

The presence of a high content of fibrous protein in connective tissue has led to considerable interest in the possible role of proteolytic enzymes in tumour invasion. Burstone (1956) and Glenner, Burstone and Meyer (1959) have shown a high activity of peptidases in the stroma of a number of primary carcinomas of several organs and more variable, but usually less, activity in the epithelium. However, only 2 cases of carcinoma of breast and 2 of " fibrocystic " disease of breast were studied. In addition Monis, Nachlas and Seligman (1959) have studied 1 carcinoma and 6 benign tumours of breast.

We have found only one reference since then to aminopeptidase activity in breast lesions and that in aspirated carcinomatous cells (Elizalde and Miller, 1967). The histochemical aminopeptidase activity in normal human breast has not been previously reported.

Histochemical demonstration of peptidase activity is limited to the demonstration of aminopeptidase, which is an enzyme hydrolysing a number of peptides which have a free $\alpha$-amino group to a terminal amino acid.

In this paper the aminopeptidase activity of normal human breast is compared with that of cystic epithelial hyperplasia, fibro-adenoma and carcinoma.

### MATERIALS AND METHODS

Breast tissue was obtained fresh from routine biopsy specimens sent for rapid diagnosis by frozen section. The age of the patients ranged from 23 to 72 years. Normal breast tissue was obtained from a portion of a radical mastectomy far removed from the main lesion. Samples were frozen immediately with a carbon dioxide ethanol mixture (—70° C.) and sectioned without delay on a Slee cryostat at 5–7 $\mu$. Fixation and dehydration of the sections were accomplished synchronously by immersion in acetone at —20° C. for 12 hours. L-leucyl-$\beta$-naphthylamide or DL-alanyl-$\beta$-naphthylamide were used as substrate for aminopeptidase, tissue sections being incubated with each of the two substrates in all cases.

Incubation was carried out at 37° C. for 4 hours in a mixture containing:

| | |
|---|---|
| Substrate (L-leucyl- or DL-alanyl-$\beta$-naphthylamide) | 4 mg. |
| Sodium chloride 0·85 % | 4·0 ml. |
| Tris buffer pH 7·05 | 5·2 ml. |
| Distilled water | 0·8 ml. |
| Fast corinth V | 6 mg. |

Sections pre-incubated for 30 minutes in 0·05 M disodium versenate, a specific aminopeptidase inhibitor, were used as controls.

A semi-quantitative assessment of the results was made by recording the depth of colour of the reaction produced on a scale 0–+++.

### RESULTS

Both the leucyl and the alanyl substrates gave similar results in all cases and these are summarized in Table I.

TABLE I.—*Aminopeptidase Activity in Normal Breast Tissue and Tumours of Breast*

| Breast tissue | Number of cases | Aminopeptidase activity in Epithelium | Aminopeptidase activity in Stroma |
|---|---|---|---|
| Normal | | | |
| (Premenopausal) | 3 | +++ | + |
| (Postmenopausal) | 3 | +++ | 0–+ |
| Carcinoma | 12 | 0–++* | 0–+ |
| Fibroadenoma | 4 | +++ | ++ |
| Cystic epithelial hyperplasia | 8 | +++ | +–++ |

* Of the 12 carcinomas the gradings were: 4–0; 7–+; and 1–++.

Normal breast tissue from both pre- and post-menopausal women, cystic epithelial hyperplasia and fibroadenomas (Fig. 1) showed marked epithelial enzyme activity whereas in carcinomas epithelial activity was less marked and more variable (Fig. 2). All the cases of carcinoma were histologically of spheroidal cell type with varying degrees of stromal fibrosis. Stromal activity was less pronounced than that of the epithelium in all instances, and most marked in fibroadenomas where it tended to be associated with cells showing high activity, including macrophages and mast cells. In carcinomas no increased stromal activity was seen adjacent to invading epithelium.

All sections pre-incubated in versenate showed absent or very faint staining.

### DISCUSSION

In the past, when L-leucyl-$\beta$-naphthylamide was used as substrate for the demonstration of aminopeptidases, it was frequently assumed that this substrate was split only by " leucine " aminopeptidase. However, similar histochemical localization is obtained with non-leucyl substrates and the enzymes demonstrated by these methods exhibit the same pattern of activation and inhibition as " leucine " aminopeptidase (Burstone, 1962). Further, more recent biochemical evidence indicates that " leucine " aminopeptidase is actually a mixture of peptidases (Patterson, Keppel and Hsiao, 1961). Both substrates showed similar enzyme activity in the same distribution although Burstone (1956) reported that the stromal elements in neoplasms were more intensely stained with the alanyl substrate. We have been unable to demonstrate any significant difference in aminopeptidase localization in the human breast with the alanyl and leucyl substrates. Accordingly the use of the term " leucine aminopeptidase " has been abandoned in favour of " aminopeptidases " when referring to enzymes hydrolysing amino acid-$\beta$-naphthylamides.

Aminopeptidase activity has been studied only to a limited extent in breast lesions and it is surprising that no one has reported the aminopeptidase activity of normal breast. We have shown strong epithelial aminopeptidase activity in normal breast but the physiological significance of this finding is not known. It is interesting that the altered hormonal blance of post-menopausal women is not associated with a change in the epithelial aminopeptidase activity in normal breast.

Carcinomas show a loss of epithelial aminopeptidase activity which varies in degree. Fibroblastic activity in general is associated with increased aminopeptidase activity (Monis *et al.*, 1959). Since stromal activity adjacent to invading carcinoma cells is the same as that of normal breast, this study offers no support to the view that aminopeptidase plays a role in the infiltration of malignant neoplasms of breast.

Burstone (1956) and Monis *et al.* (1959) reported that the epithelium of fibroadenomas and cystic glandular hyperplasia shows well-marked aminopeptidase activity. We have not only confirmed these observations but have shown that the activity is not greater than that of normal breast epithelium. Stromal activity in fibroadenomas is consistently more marked than in all other breast lesions.

### SUMMARY

Aminopeptidase activity has been studied histochemically in normal human breast and in benign and malignant lesions of breast. Benign lesions show high epithelial aminopeptidase activity of the same order as that of normal breast whereas malignant tumours usually show less and more variable activity in the epithelium. From these results there is little or no support for the view that aminopeptidase is concerned in the process of invasion of breast cancer. Stromal activity is consistently more pronounced in fibroadenomas than in all other lesions of the human breast.

This work was supported by grants to Professor A. R. Currie from the British Empire Cancer Campaign for Research and from the Scottish Hospital Endowments Research Trust. We are grateful to Mr. George D. Milne for technical help.

### REFERENCES

Burstone, M. S.—(1956) *J. natn. Cancer Inst.*, **16**, 1149.—(1962) 'Enzyme Histochemistry and Its Application in the Study of Neoplasms', New York (Academic Press) p. 406.

Elizalde, A. and Miller, S. P.—(1967) *Cancer, N.Y.*, **20**, 423.

Glenner, G. G., Burstone, M. S. and Meyer, D. B.—(1959) *J. natn. Cancer Inst.*, **23**, 857.

Monis, B., Nachlas, M. M. and Seligman, A. M.—(1959) *Cancer, N.Y.*, **12**, 601.

Patterson, E. K., Keppel, A. and Hsiao, S. H.—(1961) *J. Histochem. Cytochem.*, **9**, 609.

---

### EXPLANATION OF PLATE

Fig. 1.—Aminopeptidase activity in an acinus from a fibroadenoma of breast. $\times 500$.

Fig. 2.—Aminopeptidase activity in carcinoma of breast. Malignant epithelial cells with associated stroma. $\times 500$.